# How Fear of COVID-19 Affects the Behavioral Intention of Festival Participants—A Case of the HANFU Festival

**DOI:** 10.3390/ijerph19042133

**Published:** 2022-02-14

**Authors:** Jian Yang, Jian Ming Luo, Rui Yao

**Affiliations:** 1 School of Journalism and Communication, Guangzhou University, Guangzhou 510006, China; keeney@gzhu.edu.cn (J.Y.); yaorui@gzhu.edu.cn (R.Y.); 2 Faculty of International Tourism and Management, City University of Macau, Macau, China

**Keywords:** fear, perceived risk, perceptual evaluation, festival attitude, behavioral intention, crowding, COVID-19

## Abstract

The recovery of the tourism industry is an important issue that has attracted much attention during the COVID-19 pandemic. Sustainable and safe festival tourism is considered an effective way of aiding in the recovery of the industry. A face-to-face survey of participants in the Guangzhou Hanfu Festival was conducted to examine the relationship between fears of COVID-19, perceived risks, perceptual evaluations, festival attitudes, behavioral intentions, and crowding during this difficult time. Results clarified how fear affects behavioral intentions in festival research, and the mediation role of perceived risk and the moderation role of crowding was confirmed. A timely set of recommendations was provided to festival operators and local governments.

## 1. Introduction

Human travel has been significantly disrupted by the COVID-19 pandemic to the greatest extent since World War II [1]. The pandemic’s effects on the tourism industry not only relates to the invalidation or suspension of visas, requirements for medical certification, self-isolation or quarantine, and complete or partial blockade but also to the situation that the fear of COVID-19 has increased dramatically around the world, thereby changing people’s future travel behavior [2].

Consequently, tourism research seeks to identify appropriate solutions to mitigate these disruptive effects. Some studies have attempted to explore the feasibility of new forms of tourism during the pandemic, including untact tourism [3], travel bubbles [4], and 360 degree virtual tours [5], which are believed to help ensure travellers’ safety, health, and social distancing. Other studies have focused on domestic and protected natural areas tourism, which has been viewed as an important way to mitigate the tourism crisis and benefit residents’ physical and mental health [6,7,8,9]. With the unpredictability of pandemics on a global level, sustainable festival tourism tactics are an essential issue because festivals significantly affect tourists’ norms and pro-social intentions [10]. Positive behavioral intentions associated with the festival contribute not only to the social cohesion of the community but also to the local economy [11,12], which would benefit both the social and economic recovery from the pandemic. However, few studies on the pandemic are related to the behavioral intentions of festival participants. 

The Hanfu Festival is held every year on the third weekend of November in China, which Hanfu enthusiasts designated. The Hanfu Festival originated from a Hanfu movement around the year 2003. The Hanfu movement was launched by enthusiasts participating in public displays of Hanfu attire, forming local Hanfu communities, and organising activities about Hanfu and traditional Chinese culture [13]. The purpose of the activity is to introduce and promote Hanfu as well as to encourage the wearing of Hanfu during festivals, sacrifices, and other important occasions [14]. As a cultural heritage with more than 3000 years of recorded history, Hanfu has been transformed from a niche interest into a fashion trend [13]. However, the Hanfu Festival in 2021 has been cancelled in many cities because of the effects of the COVID-19 pandemic. According to the Chinese Health Department, on 21 November 2021, there were five high-risk areas and 62 medium-risk areas in Mainland China (wsjkw.gd.gov.cn/xxgzbdfk/fkdt/content/post_3668356.html (in Chinese), accessed on 12 January 2022). Guangzhou is one of the few cities that can hold the Hanfu Festival as scheduled. The Guangzhou Hanfu Festival will undoubtedly provide opportunities for festival research in the context of COVID-19. 

According to Rather [15], fear of COVID-19 and perceived risk play important roles in influencing attitude and behavioral intention. Zajonc’s [16] theories suggested that researchers should consider cognition and emotion to work independently during mental processing. However, few studies have verified the relationship and difference between cognition and emotion. Additionally, because of the social distancing measures, most of the tourism research related to COVID-19 is based on online survey data, and the respondents are only considering travelling [3,4,10,15,17,18]. Few studies can evaluate behavioral intentions after participation in festive events during the pandemic [19].

Therefore, to fill these two gaps in festival theory, the primary research question of this study is how COVID-19 influences behavioral intention in domestic festivals from an emotional and cognitive perspective, and what are the differences between the two perspectives.

In order to conclude this study, the remaining sections are arranged as follows: Hypotheses development is presented in the Literature Review section. Methodology and respondent profile are summarised in the Research Method section. Data analysis is shown in the Results section. Under the section Discussion and Conclusions, the findings are discussed and concluded. Finally, theoretical implications, recommendations, and suggestions to festivals operators and local governments are provided.

## 2. Literature Review

### 2.1. Fear of COVID-19

Fear is defined as a primary, intense emotion that is triggered when a person detects an imminent threat, causing a series of physiological changes and an immediate alarm in the body [20,21]. Fear is a result of interactions in which actors are subjected to the power of others, which is greater than their own [22]. For years, the outbreak of diseases or pandemics has been considered a source of fear [23]. Because fear stems from perceived threats, the intensity of fear and concern about COVID-19 can indicate the perceived threat of the virus [24].

Vaccines are one of the most efficient ways to prevent the spread of infectious illnesses [25]. However, the vast number of illnesses and deaths associated with COVID-19 vaccination may have heightened risk perception and fear [26,27]. The effect of fear is not only statistically but also economically significant, and city-level data across countries indicate fear is the primary reason for a fall in mobility [28]. To make matters worse, the sharing of fears and observing the behavior of others has resulted in a significant increase in fear of COVID-19 and spread among individuals [29]. 

Current research explored how fear affects individuals’ behavior and attitudes to reduce the effects of fear on COVID-19. Yildirim and Guler [30] believe that under the influence of the COVID-19 pandemic, the individual’s perceived risk is affected by psychological conditions, such as fear and worry because the emotional dimension of perceived risk is related to the individual’s worries and fears on the experience of potential threats [31]. Thus, a person’s fear of COVID-19 could significantly predict their risk perception [32].

For people who are considering travelling, intentions and attitudes toward travel are negatively affected by the fear of COVID-19 [4,15], which negatively moderates the link between intention to revisit and its antecedents, such as customer brand engagement, brand co-creation, and destination reputation [2,33]. However, the effects of fear on behavior and attitude are not static, and varying degrees of fear may have opposite effects [34,35]. People who are feeling fear have a sense of uncertainty and lack of control and need to experience certainty and control that makes them avoid risk [36,37]. Based on this theory, the perception and attitudes of people who have participated in scheduled festivals are likely to be positive, since the scheduled events compared with festivals that have been suspended because of the pandemic offer more certainty.

The following hypotheses are derived from the previous literature:

**H1.**  *Fear of COVID-19 is positively associated with perceived risk.*

**H2.**  *Fear of COVID-19 is positively associated with perceptual evaluation.*

**H3.**  *Fear of COVID-19 is positively associated with festival attitude.*

### 2.2. Perceived Risk

Perceived risk in this research is a cognitive variable, as perceived risk represents the cognitive probability of being exposed to threats and dangers [38]. Perceived risk can be defined as the subjective belief that a loss may occur when attempting to achieve desired outcomes through a product or service [39,40]. Particularly, perceived risk in tourism refers to situations that clearly determine whether a traveller will choose to avoid specific destinations [41]. As a result, perceived risk in tourism is associated mainly with the tourist’s uncertainty and is influenced by the inherent subjective biases informed by the potential adverse consequences of tourism consumption [42,43,44]. 

Previous research has verified that perceived risk is determined by the perceived danger of travelling [45]. If the perceived risk exceeds an individual’s acceptable level, then they may change their minds about travelling [46]. Most travellers will change their travel plans if a destination is deemed to be a high risk [47]. These potential dangers during travel are often caused by terrorism, psychological, or natural disaster [41,48,49]. In particular, in the face of the variant virus of COVID-19, even if vaccination is widely carried out globally, the global epidemic is still not under control, and tourists will feel a higher risk. 

Because the perceived risk of tourists is likely to have a considerable effect on their satisfaction and attitude toward tourism [3,18,50], the possibility of a negative evaluation can be increased by unexpected perceived risk [51]. Thus, as part of COVID-19 research, the risk associated with festivals also needs to be discussed in conjunction with evaluation and attitudes. It is important for festival organisers and marketers to minimise negative evaluations and attitudes during the recovery of the travel market.

The following two hypotheses are derived from the previous literature:

**H4.**  *Perceived risk is negatively associated with perceptual evaluation.*

**H5.**  *Perceived risk is negatively associated with festival attitude.*

### 2.3. Perceptual Evaluation, Festival Attitude, and Behavioral Intention

Davis [52] suggested that festivals cannot be evaluated in isolation without considering their geographic location. Because perceptions of a place are influenced mainly by images presented before and during the visit [53], festival research on perception evaluation is often carried out with destination-related theories, such as place identity, place dependence, and place image [54,55]. Therefore, perceptual evaluation in this research is defined as the process of understanding a festival based on beliefs and knowledge of the host place [56]. Previous studies confirmed that perception evaluation was associated closely with consumer behavior and marketing studies in the tourism industry [57]. A tourist’s perceptual evaluation of a destination is a good determinant of emotional evaluation and overall image [56,58,59]. Behavioral intention and satisfaction are negatively correlated with the festival’s negative evaluation [60,61]. Positive perceptual evaluations can enhance engagement and interactions among diverse groups, and even enrich communal livability [62].

The term attitude describes an enduring combination of beliefs around a situation or object that predisposes one to respond in a preferred manner [63]. As part of the overall attitude measurement, consumers are interrogated using a wide range of information to determine their beliefs and attributes about a particular subject [64]. A person’s overall satisfaction with the festival can be defined as their attitude toward the festival as a whole [65]. It has been suggested that attitudes toward festivals can be divided into cognitive and affective attitudes, corresponding to festival quality and satisfaction, respectively [66]. The attitude of a tourist is considered to be an important factor in determining their behavior, especially in the research based on the theory of planned behavior [67,68].

Intention refers to a determination to take a specific action in the future and represents the probability of putting one’s beliefs into practice [69]. Tourism studies generally believe a tourist’s behavioral intention can be measured by their visit to the destination again or by recommending it to others [70,71]. In this study, behavioral intentions refer to visitors’ intentions to participate in the Hanfu festival again or recommend it to others. A well-established relationship exists between attitude and intention in marketing literature [72]. Behavioral intentions are determined by an individual’s overall attitude toward an object [67,73]. Numerous studies in the tourism field have established a positive correlation between attitudes and behavior [74,75,76,77]. It is believed that travellers’ behavioral intentions, which can be used to predict visitors’ actual behavior and predict the future behavior of tourists [78,79], indicate the successful development of a destination [80] and maintain it [81,82].

The following three hypotheses are derived from the previous literature:

**H6.**  *Perceptual evaluation is positively associated with festival attitude.*

**H7.**  *Perceptual evaluation is positively associated with behavioral intention.*

**H8.**  *Festival attitude is positively associated with behavioral intention.*

### 2.4. Crowding

Crowding is defined as a pressure situation caused by a limitation on space, but these limitations do not account for all factors that influence the perception of crowding because this perception may also be driven by psychological variables [83]. In general, crowding of destinations would hurt tourists’ emotions, attitude, destination appraisal, attractiveness, activity safety, and festival experience [84,85,86,87,88]. Problems are likely to arise, especially for festivals with subsequent dense crowding [89]. Crowding is an important issue when organising risk and safety management in festivals [90].

In response to the COVID-19 pandemic, social distancing measures have been adopted around the globe because COVID-19 incidence and mortality decreased by 26% and 31%, respectively, with each unit increase in social distance [91]. Consequently, in the post-COVID-19 period, tourists will be more sensitive to crowding and prefer to avoid crowded areas in the short run [92]. Psychological theories suggest that this phenomenon occurs because the behavioral immune system is activated, and people feel negatively affected by crowded environments and perceive them as dangerous, which is considered an adaptive method of avoiding disease [93]. Because people pay attention to their physical vulnerabilities which activate people’s deep-rooted evolutionary protection mechanisms, perceived COVID-19 infectability has a significant psychological effect on tourists’ perception of crowding [17]. Given Albayrak et al. [94], crowding moderates the link between tourist emotional responses and attitude.

The three hypotheses are derived from the previous literature:

**H9.**  *Crowding moderates the relationship between fear of COVID-19 and perceived risk.*

**H10.**  *Crowding moderates the relationship between fear of COVID-19 and perceptual evaluation.*

**H11.**  *Crowding moderates the relationship between fear of COVID-19 and festival attitude.*

## 3. Research Methods

**Figure 1:**
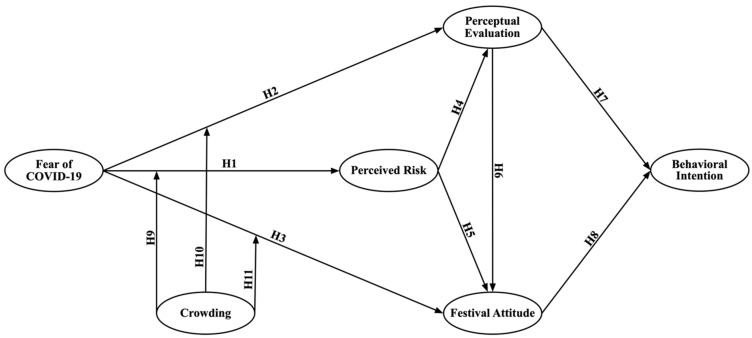
Conceptual framework.

Referring to the hypotheses developed in the previous section, a research model with fear of COVID-19 as the independent variable and behavioral intention as the dependent variable is presented in Figure 1.

The quantitative research method was used in this study. The previous research suggested that the PLS-SEM has been demonstrated to be effective for complex or exploratory research models [95]. Further, PLS-SEM can be used to analyse the moderator’s influence on the relationship between two constructs [96]. This research model consists of 8 direct influence hypotheses and 3 moderating hypotheses. The main research objective is to explore the fear of COVID-19 in festival research and how it can predict behavioral intentions. Therefore, the PLS-SEM is a more suitable method for this research. This research used SmartPLS 3 for research model analysis. The entire data analysis process was completed in two parts, including Measurement Model Evaluation and Structure Model Evaluation [97].

### 3.1. Research Instrument

In order to collect data for this quantitative study, questionnaires were used. The questionnaire was developed based on measurement scales from existing research. A seven-point Likert scale was employed in this study. Back-translation was adopted because the measurement items were written in English [98]. Bilingual tourism scholars verified the translation. Items with unsatisfactory loading values were eliminated. The questionnaire and item details can be found in Table 1.

### 3.2. Data Collection and Respondent Profile

The 2021 Guangzhou Hanfu festival was held at Haizhu Lake Park from 19 to 21 November 2021. Since Guangzhou was a non-pandemic area during the period of the Guangzhou Hanfu Festival, this study obtained a safe face-to-face survey scenario. The data collection was carried out by using a field survey. The survey data was closer to the travel behavioral intentions in the post-pandemic period.

In these three days, 22 well-trained assistants distributed the questionnaire to visitors who had participated in the Hanfu Festival. Each research assistant had a tablet for data collection. In order to collect enough samples without disturbing visitor’s participation in the Hanfu Festival, our research selected multiple sites inside and outside the park for data collection, such as festival hotspots, exits, nearby bus stops, and the Datang subway station (the only subway station near Haizhu Lake Park). A convenience sampling method was used in this study. A filter question was used to ensure that the respondents had participated in the Hanfu Festival. A total of 358 valid questionnaires were obtained after excluding incomplete questionnaires and those with flatlining responses.

Table 2 summarises the respondents’ demographic information collected during the formal investigation stage, including gender, age, educational background, and monthly income. The gender distribution is approximately equal. There were 48.6% male respondents and 51.4% female respondents. A number of respondents had ages between 18 to 40 years old and had an undergraduate degree. 

## 4. Results

This section presents the results of the research model analysis performed by SmartPLS 3. According to Hair Jr., Hult, Ringle, and Sarstedt [97], this section first presents the evaluation results of the measurement model, followed by the structural model evaluation results, and the results of moderating and mediating effect as supplements to the structural model evaluation.

### 4.1. Measurement Model Evaluation

The evaluation results in this section are mainly used to show that the measurement model has reliability, convergent validity, and discriminant validity.

Table 3 demonstrates the result of reliability and convergent validity. The Cronbach’s α values ranging from 0.763 to 0.888 are above 0.7. The CR values fall in a reasonable range, between 0.862 and 0.93. Thus, the internal consistency reliability of the measurement model is established [103,104].

Both of the factor loadings ranging from 0.746 to 0.926 and AVE values ranging from 0.586 to 0.817 are above the threshold [97,105]. Thus, the convergent validity of the measurement model can be confirmed.

The results of the Fornell-Larcker criterion and HTMT analysis demonstrate that the measurement model has discriminant validity. Table 4 shows the satisfactory discriminant validity. It can be seen from bold fonts that the square roots of AVEs on each construct are greater than the correlations between constructs [106]. All HTMT ratios were below 0.85, ranging from 0.836 to 0.084, again with satisfactory results [107]. 

### 4.2. Structure Model Evaluation

Table 5 shows the value of the determination coefficient (R^2^) and predictive correlation (Q^2^). R^2^ values, which are between 0.268 and 0.651, confirmed that all external variables have a satisfactory impact on internal dependent variables [95]. All Q^2^ evaluation results are above 0, indicating that the structural model in this study is capable of predicting the data accurately [108,109].

**Figure 2:**
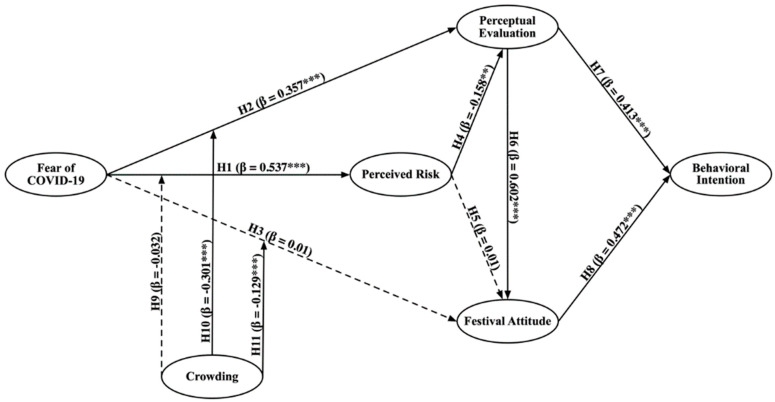
Results of the hypotheses (Note: *** *p* value < 0.001;** *p* value < 0.01).

The statistical significance between variables was tested using the bootstrapping resampling method (5000 resamples). The results can be found in Table 6 and Figure 2. Fear of COVID-19 has a significant relationship with perceived risk (β = 0.537, t = 15.034, *p* < 0.001) and perceptual evaluation (β = 0.357, t = 6.276, *p* < 0.001), thus supporting H1 and H2. H4 is also supported because perceived risk is significantly related to perceptual evaluation (β = −0.158, t = 2.652, *p* < 0.01). The path coefficients of perceptual evaluation on festival attitude and behavioral intention are 0.602 (t = 14.331, *p* < 0.001) and 0.413 (t = 7.926, *p* < 0.001), respectively. The path coefficient from festival attitude on the behavioral intention is 0.472 (t = 9.08, *p* < 0.001). These results support H6, H7, and H8. 

In addition to the hypothesis testing, the *f*^2^ values are used for the supplementary analysis of the hypothesis quality. The *f*^2^ values of all supported hypotheses shown in Table 6 are over 0.02, and thus, various exogenous factors have a significant effect on their corresponding endogenous factors [97]. Moreover, in Table 6, all VIFs are below five, which ranged from 1.000 to 1.773, indicating the absence of the issue of multicollinearity [91].

### 4.3. Moderating Effect

**Figure 3:**
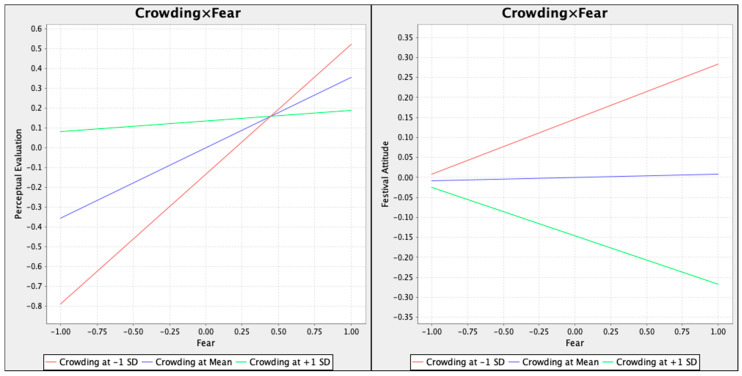
Simple slope test.

The interactive effect of crowding and fear of COVID-19 are significantly associated with perceptual evaluation (β = −0.301, t = 6.599) and festival attitude (β = −0.129, t = 4.735), suggesting that the moderating effect of crowding is supported, and thus, H10 and H11 are supported. The results of the simple slope analysis (Figure 3) demonstrate that participants who perceived high crowding in the Hanfu festival have lower perceptual evaluation and festival attitude when they perceive the same level of fear. Because the lower crowding line has a steeper slope, the moderation effect of crowding is negative.

### 4.4. Mediation Effect

The results of the mediation analysis of perceived risk, perception evaluation, and festival attitude can be found in Table 7. Because H3 and H5 are rejected, the result indicates that perceptual evaluation acts as a complete mediation role between fear of COVID-19 and festival attitude (β = 0.215, t = 5.704, *p* < 0.001) and between perceived risk and festival attitude (β = −0.093, t = 2.614, *p* < 0.01). Results also suggest that fear of COVID-19 influenced behavioral intention through perceived risk, perceptual evaluation, and festival attitude. A comparison of the path coefficients in the mediation test indicates that perceived risk is the critical variable determining whether path coefficients are positive or negative. The indirect path through perceived risk are negative (β = −0.024, t = 2.458, *p* < 0.05 and β = −0.035, t = 2.368, *p* < 0.05), respectively, and the path without perceived risk are positive (β = 0.101, t = 5.071, *p* < 0.001 and β = 0.147, t = 4.588, *p* < 0.001).

## 5. Discussion

In the wake of the WHO’s announcement that COVID-19 is a pandemic, many people realised that they were at risk for this deadly disease [110], and fear spread in human society. According to the results of this study, fear of COVID-19 does not necessarily negatively affect the intention to participate in the Hanfu festival, and the critical negative factor is participants’ perceived risk. This study finds that perceptual evaluation and festival attitude contribute to increased behavioral intention in festival participants. Fear of COVID-19 has a direct positive effect on perceptual evaluation and perceived risk, but the perceived risk has a direct negative impact on perceptual evaluation. Although fear of COVID-19 and perceived risk do not directly impact festival attitude, perceptual evaluation is a complete mediation factor in these two relationships. Crowding plays a moderating role in the relationship between fear of COVID-19 and perceptual evaluation and between fear of COVID-19 and festival attitude. In conclusion, Hypotheses 1, 2, 4, 6–8, 10 and 11 are supported, while Hypotheses 3, 5 and 9 are not.

The findings were in line with those reported in the literature. Fear of COVID-19 is positively correlated with both perceived risk and perceptual evaluation toward the festival (H1 and H2). The results reinforce previous literature [32,36,37] but contradict the statement of Rather [15] and Luo and Lam [4]. One possible explanation is that this study focused on people who took part in a festival, whereas respondents of previous studies only assumed they would travel. The positive effect of perceived risk on perceptual evaluation (H4) and the relationship between perceptual evaluation, attitude, and behavioral intention (H6 to 9) confirm previous studies’ findings [51,68,75,111]. 

The mediating test confirmed the reason H3 and H5 are rejected. Fear of COVID-19 and perceived risk indirectly affect festival attitude via perceptual evaluation, which aligns with the findings of previous studies [51]. This study also introduced perceived risk, perceptual evaluation, and festival attitude as mediators between the fear of COVID-19 and festival participating intention. Perceived risk is an essential variable influencing whether fear of COVID-19 is positively or negatively correlated with behavioral intention.

As an individual value, the crowding perception of festival participants has changed considerably in the context of the pandemic. In this study, crowding negatively moderates the link between fear of COVID-19 and perceptual evaluation (H10) and the connection between fear of COVID-19 and festival attitude (H11), meaning that the higher crowding perception, the weaker the influence of fear of COVID-19 on perceptual evaluation and festival attitude. However, this scenario does not apply to the effects of perceived risk (H9). A possible explanation for this result is that many factors jointly affect the perceived risk of participants, and the perception of crowding is not the most important factor.

## 6. Conclusions and Future Research

### 6.1. Theoretical Implications

Literature indicated that fear of COVID-19 is not just a variable that negatively affects behavioral intentions. Even if individuals are in fear of COVID-19, they can have a positive effect on behavioral intention without the influence of perceived risk. This study expands the understanding of the mediating role of perceived risk and reinforces the theories that fear can be a powerful, persuasive strategy, but it can also backfire when misused [35]. These results lend support to the theory that cognition and emotion are independent during mental processing [16,112]. The findings also can explain why emotions have different effects on behavioral intention.

In this study, several mediators are introduced to assess the indirect effects of fear of COVID-19 on behavioral intention. Our results verified the mediating effects of perceived risk, perceptual evaluation, and festival attitude. In addition to perceived risk, perceptual evaluation is another important mediating variable. Because perceptual evaluation is related closely to the place where the festival is held, this study shows that an individual’s attitude toward festivals comes from an individual’s evaluation of the relationship between festivals and host places. 

In the context of the pandemic, the perception of crowding becomes a sensitive factor affecting individual attitudes and behaviors. In previous studies, crowding has been studied primarily from a direct effect perspective [85,86], and this study is the pioneer in showing how crowding moderates the relationship between emotion and attitude. The result contributes to the literature that the positive value of holding a festival during a pandemic, which is very fragile, can be offset by crowding perception. The theory of protection mechanisms proposed by previous studies [17,93] continues to play a role in tourists’ attitudes during and after the pandemic.

### 6.2. Practical Implications

The study provides valuable insight into how fear affects the intentions of festival participants on a practical level. An important finding of the study is that the fear of the virus does not undermine an individual’s behavioral intention to participate in festivals, while perceived risk does. Festival operators and related organisers should not ignore the tourists’ demand for local festivals and events during the pandemic. Suppose festival operators and related organisers can put risk reduction measures in place and meet the needs of potential visitors during the pandemic. In that case, the festival can still receive a positive response from participants despite the considerable personal fears that the two-year-long COVID-19 pandemic has caused.

Therefore, festival operators and related organisers should first consider measures to reduce the perceived risks of participants. Festival organisers should strengthen cooperation with service personnel to jointly formulate service specifications during the pandemic, such as requiring service personnel and performers to be vaccinated and submit virus test certificates, requiring the wearing of masks throughout the event, and ensuring that the facilities used by crowds are cleaned. At the same time, non-contact services or self-service (such as participation by appointment, online ticketing, or robot sales) should be expanded. For festival participants, festival organisers should consider introducing epidemic prevention knowledge and health protection measures through broadcast at the event site, such as reminding visitors to wear masks, maintain safe social distancing, use hand sanitiser, verify visitors’ healthy travel code at the entrance, and monitor visitors’ body temperature through infrared thermography. Since the pandemic affects personal crowding perception, festival operators should consider organising festivals and events outdoors or in an open place during the pandemic. Smaller community festivals may be better suited, as these types of festivals are more likely to limit attendance and reduce festival host and attendee costs [113]. Participants’ itineraries need to be designed carefully. A layout with scattered hot spots can avoid excessive aggregation of personnel. A mobile app can be used to inform participants of the waiting time at the hotspot so that participants can arrange the tour reasonably.

This research has verified festival activities during the pandemic produced positive evaluations and are beneficial to the destination city. Therefore, the government should control the pandemic as soon as possible and create a safe social environment. When the epidemic is under control, the government should take a positive attitude towards holding the festival activities and provide convenience for festival operators. During the festival, the local government should manage the flow of people outside the festival venue, ensure smooth traffic in the surrounding areas of the festival, and strengthen public transportation services. The future of public health is likely to become increasingly digital [114]. In order to build public trust and reduce risk perceptions for individuals involved in festival activities, governments are encouraged to use digital technologies to create safe community environments and strong communication strategies, such as contact tracing and epidemiological intelligence.

### 6.3. Limitations and Future Research

Our research data were collected at a specific time. Fears vary depending on vaccine effectiveness, vaccination rates, and the use of specific drugs. Different countries also have different social distancing restrictions, which affect individuals’ risk perception of participating in festivals. Consequently, in the future, the research model can be tested on samples from different countries and regions to determine its relevance under different circumstances. Furthermore, factors such as individual differences, time and space, and the crowding environment can be regarded as antecedents of perceived crowding [86]. Future studies should consider comparing different types of participants (such as vaccinated and unvaccinated individuals, young and old, male and female) and festivals (such as held day and night, indoor and outdoor). The constructs in our research are limited, and some constructs affect each other in our research models, such as the relationship between perceived risk and fear [115,116]. Therefore, the research model can be enhanced by incorporating other constructs in the future.

## Figures and Tables

**Table 1:** Measurement Items and Study Constructs.

Constructs	Measurement Items	Previous Study
Fear of COVID-19 (FCV)	FCV1: I am most afraid of the novel coronavirus. FCV2: It makes me uncomfortable to think about the novel coronavirus. FCV3: I am afraid of losing my life because of COVID-19. FCV4: When watching news and stories about the novel coronavirus on social media or any other media (i.e., TV, radio), I become nervous or anxious. FCV5: I cannot sleep because I am worried about being infected with the novel coronavirus.	Ahorsu, Lin, Imani, Saffari, Griffiths and Pakpour [29]
Perceived Risk (PRK)	PRK1: Given the current situation, I prefer to avoid attending large festival activities. PRK2: Given the current situation, I prefer to shorten the duration of my attendance in festival activities. PRK3: I feel more averse to attending festival activities because of the risk from COVID 19.	Karl [49]; Sánchez-Cañizares, et al. [99]
Festival Attitude (FA)	FA1: Hanfu Festival is useful. FA2: Hanfu Festival is valuable. FA3: Hanfu Festival is beneficial. FA4: Hanfu Festival is attractive.	Rather [15]; Bae and Chang [3]
Perceptual Evaluation (PE)	PE1: Hanfu Festival made Guangzhou more attractive as a tourist destination. PE2: Hanfu Festival increased the competitiveness of Guangzhou as a tourist destination. PE3: Hanfu Festival contributed to the improvement of the regional image.	Ross [100]; Baloglu and McCleary [56]
Behavioral Intention (BI)	BI1: I will recommend the Hanfu Festival to others. BI2: I will talk about the Hanfu Festival positively. BI3: I will revisit the Hanfu Festival.	Chen and Tsai [101]; Chi and Qu [102]
Crowding (CD)	CD1: People are very close to me in the Hanfu Festival area. CD2: There are many people in the Hanfu Festival area. CD3: The Hanfu Festival area is crowded. CD4: The rest areas are crowded in Hanfu Festival.	Yin, Cheng, Bi and Ni [86]; Liu and Ma [85]

**Table 2:** Descriptive summary of sociodemographic profile.

Demographic	Categories	Frequency	Percentage
Gender	Male	174	48.6%
	Female	184	51.4%
Age	18–30	224	62.6%
	31–40	112	31.3%
	41–50	16	4.5%
	51–60	4	1.1%
	Over 60	2	0.5%
Education	High school or below	59	16.5%
	Diploma	105	29.3%
	Undergraduates	175	48.9%
	Graduates or above	19	5.3%
Income	RMB 5000 or below	91	25.4%
	RMB 5001–10,000	140	39.1%
	RMB 10,001–20,000	102	28.5%
	RMB 20,001–30,000	19	5.3%
	RMB 30,001 or above	6	1.7%

**Table 3:** Result of reliability and convergent validity.

Constructs	Measured Item	Factor Loading	Cronbach’s Alpha	CR	AVE
Fear of COVID-19 (FCV)			0.824	0.876	0.586
	FCV1	0.782			
	FCV2	0.746			
	FCV3	0.753			
	FCV4	0.751			
	FCV5	0.793			
Perceived Risk (PRK)			0.763	0.862	0.675
	PRK1	0.833			
	PRK2	0.827			
	PRK3	0.806			
Festival Attitude (FA)			0.874	0.913	0.726
	FA1	0.838			
	FA2	0.895			
	FA3	0.856			
	FA4	0.815			
Perceptual Evaluation (PE)			0.871	0.921	0.795
	PE1	0.907			
	PE2	0.900			
	PE3	0.867			
Behavioral Intention (BI)			0.888	0.93	0.817
	BI1	0.893			
	BI2	0.913			
	BI3	0.906			
Crowding (CD)			0.882	0.907	0.711
	CD1	0.926			
	CD2	0.856			
	CD3	0.793			
	CD4	0.791			

**Table 4:** Fornell–Larcker criterion and HTMT analysis.

	FCV	PRK	FA	PE	BI	CD
Fear of COVID-19 (FCV)	**0.765**	0.647	0.244	0.394	0.273	0.197
Perceived Risk (PRK)	0.534	**0.822**	0.084	0.085	0.074	0.160
Festival Attitude (FA)	0.213	0.058	**0.852**	0.741	0.836	0.175
Perceptual Evaluation (PE)	0.337	0.053	0.66	**0.891**	0.821	0.167
Behavioral intention (BI)	0.228	0.02	0.745	0.725	**0.904**	0.092
Crowding (CD)	0.182	0.025	−0.024	0.198	0.067	**0.843**

Note: Bold fonts are the square root of the AVE. The values above the bold fonts are the HTMT ratios. The values below the bold fonts are estimated correlations.

**Table 5:** R^2^ and Q^2^.

Latent Variable	R^2^	Q^2^
Festival Attitude	0.504	0.348
Perceived Risk	0.293	0.182
Perceptual Evaluation	0.268	0.196
Revisit Intention	0.651	0.525

**Table 6:** Results of hypotheses analysis.

Hypothesis and Paths	β-Values	t-Values	*p*-Values	f-Square	VIF	Result
**H1:** *Fear of COVID-19 → Perceived Risk*						
	0.537	15.034	0.000	0.405	1.000	Accept
**H2:** *Fear of COVID-19 → Perceptual Evaluation*						
	0.357	6.276	0.000	0.117	1.480	Accept
**H3:** *Fear of COVID-19 → Festival Attitude*						
	0.010	0.200	0.841	0.000	1.656	Reject
**H4:** *Perceived Risk → Perceptual Evaluation*						
	−0.158	2.652	0.008	0.024	1.416	Accept
**H5:** *Perceived Risk → Festival Attitude*						
	0.010	0.184	0.854	0.000	1.451	Reject
**H6:** *Perceptual Evaluation → Festival Attitude*						
	0.602	14.331	0.000	0.543	1.355	Accept
**H7:** *Perceptual Evaluation→Behavioral Intention*						
	0.413	7.926	0.000	0.276	1.773	Accept
**H8:** *Festival Attitude → Behavioral Intention*						
	0.472	9.081	0.000	0.360	1.773	Accept
**H9:** *Crowding × Fear of COVID-19 → Perceived Risk*						
	−0.032	0.484	0.628	0.004	1.021	Reject
**H10:** *Crowding × Fear of COVID-19→Perceptual Evaluation*						
	−0.301	6.599	0.000	0.159	1.016	Accept
**H11:** *Crowding × Fear of COVID-19 → Festival Attitude*						
	−0.129	4.735	0.000	0.090	1.163	Accept

**Table 7:** Results of the mediation tests.

Paths	Coefficient	t-Values	*p*-Values	Decision
*Perceived Risk → Perceptual Evaluation → Festival Attitude*				
	−0.093	2.614	0.009	Accept
*Fear of COVID-19 → Perceptual Evaluation → Festival Attitude*				
	0.215	5.704	0.000	Accept
*Fear of COVID-19 → Perceived Risk → Perceptual Evaluation → Festival Attitude → Behavioral Intention*				
	−0.024	2.458	0.014	Accept
*Fear of COVID-19 → Perceived Risk → Perceptual Evaluation → Behavioral Intention*				
	−0.035	2.368	0.018	Accept
*Fear of COVID-19 → Perceptual Evaluation → Festival Attitude → Behavioral Intention*				
	0.101	5.071	0.000	Accept
*Fear of COVID-19 → Perceptual Evaluation → Behavioral Intention*				
	0.147	4.588	0.000	Accept

## Data Availability

The data presented in this study are available on request from the corresponding author. The data are not publicly available due to privacy issues.

## References

[1] Beck M.J., Hensher D.A. (2020). Insights into the impact of COVID-19 on household travel and activities in Australia—The early days under restrictions. Transp. Policy.

[2] Hassan S.B., Soliman M. (2020). COVID-19 and repeat visitation: Assessing the role of destination social responsibility, destination reputation, holidaymakers’ trust and fear arousal. J. Destin. Mark. Manag..

[3] Bae S.Y., Chang P.-J. (2020). The effect of coronavirus disease-19 (COVID-19) risk perception on behavioural intention towards ‘untact’ tourism in South Korea during the first wave of the pandemic (March 2020). Curr. Issues Tour..

[4] Luo J.M., Lam C.F. (2020). Travel Anxiety, Risk Attitude and Travel Intentions towards “Travel Bubble” Destinations in Hong Kong: Effect of the Fear of COVID-19. Int. J. Environ. Res. Public Health.

[5] Yang T., Lai I.K.W., Fan Z.B., Mo Q.M. (2021). The impact of a 360 degrees virtual tour on the reduction of psychological stress caused by COVID-19. Technol. Soc..

[6] Volgger M., Taplin R., Aebli A. (2021). Recovery of domestic tourism during the COVID-19 pandemic: An experimental comparison of interventions. J. Hosp. Tour. Manag..

[7] Arbulú I., Razumova M., Rey-Maquieira J., Sastre F. (2021). Can domestic tourism relieve the COVID-19 tourist industry crisis? The case of Spain. J. Destin. Mark. Manag..

[8] Grima N., Corcoran W., Hill-James C., Langton B., Sommer H., Fisher B. (2020). The importance of urban natural areas and urban ecosystem services during the COVID-19 pandemic. PLoS ONE.

[9] Som A.P.M., Mohamed B., Yew W.K. (2006). Tourism in protected areas: Constraints and challenges. TEAM J. Hosp. Tour..

[10] Chi X., Cai G., Han H. (2021). Festival travellers’ pro-social and protective behaviours against COVID-19 in the time of pandemic. Curr. Issues Tour..

[11] Rao V. (2001). Celebrations as social investments: Festival expenditures, unit price variation and social status in rural India. J. Dev. Stud..

[12] Thrane C. (2002). Jazz Festival Visitors and Their Expenditures: Linking Spending Patterns to Musical Interest. J. Travel Res..

[13] Wang X., Colbert F., Legoux R. (2020). From Niche Interest to Fashion Trend: Hanfu Clothing as a Rising Industry in China. Int. J. Arts Manag..

[14] Xiaodie P., Haixia Z., Yongfei Z. (2020). An Analysis of the Current Situation of the Chinese Clothing Craze in the Context of the Rejuvenation of Chinese Culture. Proceedings of the 2020 4th International Seminar on Education, Management and Social Sciences (ISEMSS 2020).

[15] Rather R.A. (2021). Monitoring the impacts of tourism-based social media, risk perception and fear on tourist’s attitude and revisiting behaviour in the wake of COVID-19 pandemic. Curr. Issues Tour..

[16] Zajonc R.B. (2000). Feeling and thinking: Closing the debate over the independence of affect. Feeling and Thinking: The role of Affect in Social Cognition.

[17] Kock F., Norfelt A., Josiassen A., Assaf A.G., Tsionas M.G. (2020). Understanding the COVID-19 tourist psyche: The Evolutionary Tourism Paradigm. Ann. Tour. Res..

[18] Yu J., Lee K., Hyun S.S. (2021). Understanding the influence of the perceived risk of the coronavirus disease (COVID-19) on the post-traumatic stress disorder and revisit intention of hotel guests. J. Hosp. Tour. Manag..

[19] Wang X., Lai I.K.W., Zhou Q., Pang Y.H. (2021). Regional Travel as an Alternative Form of Tourism during the COVID-19 Pandemic: Impacts of a Low-Risk Perception and Perceived Benefits. Int. J. Environ. Res. Public Health.

[20] Moyle B.D., Moyle C.-L., Bec A., Scott N. (2019). The next frontier in tourism emotion research. Curr. Issues Tour..

[21] Cori L., Curzio O., Adorni F., Prinelli F., Noale M., Trevisan C., Fortunato L., Giacomelli A., Bianchi F. (2021). Fear of COVID-19 for Individuals and Family Members: Indications from the National Cross-Sectional Study of the EPICOVID19 Web-Based Survey. Int. J. Environ. Res. Public Health.

[22] Kemper T.D. (1987). How Many Emotions Are There? Wedding the Social and the Autonomic Components. Am. J. Sociol..

[23] Moukaddam N. (2019). Fear, soutbreaks, and pandemics: Lessons learned. Psychiatry Times.

[24] Sloan M.M., Haner M., Graham A., Cullen F.T., Pickett J.T., Jonson C.L. (2021). Pandemic emotions: The extent, correlates, and mental health consequences of fear of COVID-19. Sociol. Spectr..

[25] Centers for Disease Control and Prevention (2011). Ten great public health achievements—United States, 2001–2010. Morb. Mortal. Wkly. Rep..

[26] Dryhurst S., Schneider C.R., Kerr J., Freeman A.L.J., Recchia G., van der Bles A.M., Spiegelhalter D., van der Linden S. (2020). Risk perceptions of COVID-19 around the world. J. Risk Res..

[27] Chu H., Liu S. (2021). Integrating health behavior theories to predict American’s intention to receive a COVID-19 vaccine. Patient Educ. Couns..

[28] Alfaro L., Faia E., Lamersdorf N., Saidi F. (2020). Social Interactions in Pandemics: Fear, Altruism, and Reciprocity.

[29] Ahorsu D.K., Lin C.-Y., Imani V., Saffari M., Griffiths M.D., Pakpour A.H. (2020). The Fear of COVID-19 Scale: Development and Initial Validation. Int. J. Ment. Health Addict..

[30] Yıldırım M., Güler A. (2020). Factor analysis of the COVID-19 Perceived Risk Scale: A preliminary study. Death Stud..

[31] Lee J.E., Lemyre L., Krewski D. (2010). A multi-method, multi-hazard approach to explore the uniqueness of terrorism risk perceptions and worry. J. Appl. Soc. Psychol..

[32] Breakwell G.M., Jaspal R. (2020). Identity change, uncertainty and mistrust in relation to fear and risk of COVID-19. J. Risk Res..

[33] Rather R.A. (2021). Demystifying the effects of perceived risk and fear on customer engagement, co-creation and revisit intention during COVID-19: A protection motivation theory approach. J. Destin. Mark. Manag..

[34] Liu S., Yang J.Z., Chu H. (2019). When we increase fear, do we dampen hope? Using narrative persuasion to promote human papillomavirus vaccination in China. J. Health Psychol..

[35] Witte K. (1994). Fear control and danger control: A test of the extended parallel process model (EPPM). Commun. Monogr..

[36] Lerner J.S., Li Y., Valdesolo P., Kassam K.S. (2015). Emotion and decision making. Annu. Rev. Psychol..

[37] Gómez-Díaz L. (2021). Destination Image in the COVID-19 Crisis: How to Mitigate the Effect of Negative Emotions, Developing Tourism Strategies for Ethnocentric and Cosmopolitan Consumers. Multidiscip. Bus. Rev..

[38] Reisinger Y., Mavondo F. (2005). Travel Anxiety and Intentions to Travel Internationally: Implications of Travel Risk Perception. J. Travel Res..

[39] Pavlou P.A., Gefen D. (2004). Building Effective Online Marketplaces with Institution-Based Trust. Inf. Syst. Res..

[40] Tseng S.-Y., Wang C.-N. (2016). Perceived risk influence on dual-route information adoption processes on travel websites. J. Bus. Res..

[41] Sönmez S.F., Graefe A.R. (1998). Influence of terrorism risk on foreign tourism decisions. Ann. Tour. Res..

[42] Adam I. (2015). Backpackers’ risk perceptions and risk reduction strategies in Ghana. Tour. Manag..

[43] Hasan M.K., Ismail A.R., Islam M.F. (2017). Tourist risk perceptions and revisit intention: A critical review of literature. Cogent Bus. Manag..

[44] Liu B., Schroeder A., Pennington-Gray L., Farajat S.A. (2016). Source market perceptions: How risky is Jordan to travel to?. J. Destin. Mark. Manag..

[45] Fuchs G., Reichel A. (2011). An exploratory inquiry into destination risk perceptions and risk reduction strategies of first time vs. repeat visitors to a highly volatile destination. Tour. Manag..

[46] Fuchs G., Reichel A. (2006). Tourist Destination Risk Perception: The Case of Israel. J. Hosp. Leis. Mark..

[47] Lepp A., Gibson H., Lane C. (2011). Image and perceived risk: A study of Uganda and its official tourism website. Tour. Manag..

[48] Carballo R.R., Leon C.J., Carballo M.M. (2017). The perception of risk by international travellers. Worldw. Hosp. Tour. Themes.

[49] Karl M. (2016). Risk and Uncertainty in Travel Decision-Making: Tourist and Destination Perspective. J. Travel Res..

[50] Han H., Yu J., Kim W. (2019). An electric airplane: Assessing the effect of travelers’ perceived risk, attitude, and new product knowledge. J. Air Transp. Manag..

[51] Sohn H.-K., Lee T.J., Yoon Y.-S. (2016). Relationship between Perceived Risk, Evaluation, Satisfaction, and Behavioral Intention: A Case of Local-Festival Visitors. J. Travel Tour. Mark..

[52] Davis A. (2016). Experiential places or places of experience? Place identity and place attachment as mechanisms for creating festival environment. Tour. Manag..

[53] Boo S., Busser J.A. (2005). The Hierarchical Influence of Visitor Characteristics on Tourism Destination Images. J. Travel Tour. Mark..

[54] Zhang C.X., Fong L.H.N., Li S. (2019). Co-creation experience and place attachment: Festival evaluation. Int. J. Hosp. Manag..

[55] Williams M., A J Bowdin G. (2007). Festival evaluation: An exploration of seven UK arts festivals. Manag. Leis..

[56] Baloglu S., McCleary K.W. (1999). A model of destination image formation. Ann. Tour. Res..

[57] Wu H.-C., Cheng C.-C. (2017). What Drives Experiential Loyalty Toward Smart Restaurants? The Case Study of KFC in Beijing. J. Hosp. Mark. Manag..

[58] Lee I., Arcodia C. (2011). The Role of Regional Food Festivals for Destination Branding. Int. J. Tour. Res..

[59] Schofield P., Thompson K. (2007). Visitor motivation, satisfaction and behavioural intention: The 2005 Naadam Festival, Ulaanbaatar. Int. J. Tour. Res..

[60] Lee J.-S., Lee C.-K., Choi Y. (2010). Examining the Role of Emotional and Functional Values in Festival Evaluation. J. Travel Res..

[61] Cole S.T., Scott D. (2004). Examining the Mediating Role of Experience Quality in a Model of Tourist Experiences. J. Travel Tour. Mark..

[62] Agboola O.P. (2021). The significance and users’ perceptual evaluation of traditional market spaces in Nigeria. SN Soc. Sci..

[63] Rokeach M. (1968). A theory of organisation and change within value-attitude systems. J. Soc. Issues.

[64] Lee J.-M. (2019). The Affect of Family Restaurant Customer’s Experiences on Customer Satisfaction, Brand Attitude, and Revisit Intentions. Int. Converg. Manag. Assoc..

[65] Tian-Cole S., Crompton J.L., Willson V.L. (2002). An Empirical Investigation of the Relationships Between Service Quality, Satisfaction and Behavioral Intentions among Visitors to a Wildlife Refuge. J. Leis. Res..

[66] Choo H., Ahn K., Petrick J.F. (2016). An integrated model of festival revisit intentions. Int. J. Contemp. Hosp. Manag..

[67] Prayag G., Chen N., Del Chiappa G. (2017). Domestic tourists to Sardinia: Motivation, overall attitude, attachment, and behavioural intentions. Anatolia.

[68] Hadinejad A., Noghan N., Moyle B.D., Scott N., Kralj A. (2021). Future research on visitors’ attitudes to tourism destinations. Tour. Manag..

[69] Kang H.-S., Park J.-M., Lee S.-M. (2017). The Influence of Event Quality on Brand Value, Satisfaction and Recommend Intention as perceived by Local Food Event Participants: Case of Miderdok Festival in Changwon Province. Culin. Sci. Hosp. Res..

[70] Oliver R.L. (1999). Whence consumer loyalty?. J. Mark..

[71] Petrick J.F. (2004). First timers’ and repeaters’ perceived value. J. Travel Res..

[72] Fishbein M., Ajzen I. (1977). Belief, Attitude, Intention, and Behavior: An Introduction to Theory and Research. Philos. Rhetor..

[73] Ekinci Y., Dawes P.L., Massey G.R. (2008). An extended model of the antecedents and consequences of consumer satisfaction for hospitality services. Eur. J. Mark..

[74] Liu J., An K., Jang S.S. (2020). A model of tourists’ civilised behaviors: Toward sustainable coastal tourism in China. J. Destin. Mark. Manag..

[75] Ulker-Demirel E., Ciftci G. (2020). A systematic literature review of the theory of planned behavior in tourism, leisure and hospitality management research. J. Hosp. Tour. Manag..

[76] Huang S., Hsu C.H. (2009). Effects of Travel Motivation, Past Experience, Perceived Constraint, and Attitude on Revisit Intention. J. Travel Res..

[77] Lam T., Hsu C.H. (2004). Theory of Planned Behavior: Potential Travelers from China. J. Hosp. Tour. Res..

[78] Tsai C.-T. (2016). Memorable Tourist Experiences and Place Attachment When Consuming Local Food. Int. J. Tour. Res..

[79] Webb T.L., Sheeran P. (2006). Does changing behavioral intentions engender behavior change? A meta-analysis of the experimental evidence. Psychol. Bull..

[80] Prayag G., Ryan C. (2012). Antecedents of tourists’ loyalty to Mauritius: The role and influence of destination image, place attachment, personal involvement, and satisfaction. J. Travel Res..

[81] Xu Z., Zhang J. (2016). Antecedents and consequences of place attachment: A comparison of Chinese and Western urban tourists in Hangzhou, China. J. Destin. Mark. Manag..

[82] Ding H.-M., Hung K.-P. (2021). The antecedents of visitors’ flow experience and its influence on memory and behavioral intentions in the music festival context. J. Destin. Mark. Manag..

[83] Stokols D. (1972). On the distinction between density and crowding: Some implications for future research. Psychol. Rev..

[84] Li L., Zhang J., Nian S., Zhang H. (2017). Tourists’ perceptions of crowding, attractiveness, and satisfaction: A second-order structural model. Asia Pac. J. Tour. Res..

[85] Liu A., Ma E. (2019). Travel during holidays in China: Crowding’s impacts on tourists’ positive and negative affect and satisfactions. J. Hosp. Tour. Manag..

[86] Yin J., Cheng Y., Bi Y., Ni Y. (2020). Tourists perceived crowding and destination attractiveness: The moderating effects of perceived risk and experience quality. J. Destin. Mark. Manag..

[87] Tseng Y.-P., Kyle G.T., Shafer C.S., Graefe A.R., Bradle T.A., Schuett M.A. (2009). Exploring the Crowding–Satisfaction Relationship in Recreational Boating. Environ. Manag..

[88] Kim D., Lee C.-K., Sirgy M.J. (2016). Examining the Differential Impact of Human Crowding Versus Spatial Crowding on Visitor Satisfaction at a Festival. J. Travel Tour. Mark..

[89] Cheng H., Liu Q., Bi J.-W. (2021). Perceived crowding and festival experience: The moderating effect of visitor-to-visitor interaction. Tour. Manag. Perspect..

[90] Mykletun R.J. (2011). Festival Safety—Lessons Learned from the Stavanger Food Festival (the Gladmatfestival). Scand. J. Hosp. Tour..

[91] VoPham T., Weaver M.D., Adamkiewicz G., Hart J.E. (2021). Social Distancing Associations with COVID-19 Infection and Mortality Are Modified by Crowding and Socioeconomic Status. Int. J. Environ. Res. Public Health.

[92] Kainthola S., Tiwari P., Chowdhary N.R. (2021). Overtourism to Zero Tourism: Changing Tourists’ Perception of Crowding Post COVID-19. J. Spat. Organ. Dyn..

[93] Wang J., Liu-Lastres B., Ritchie B.W., Mills D.J. (2019). Travellers’ self-protections against health risks: An application of the full Protection Motivation Theory. Ann. Tour. Res..

[94] Albayrak T., Güzel Ö., Caber M., Kılıçarslan Ö., Dursun Cengizci A., Güven A. (2020). How does perceived crowding moderate tourist shopping experience and satisfaction relationship?. Int. J. Tour. Cities.

[95] Hair J.F., Ringle C.M., Sarstedt M. (2011). PLS-SEM: Indeed a Silver Bullet. J. Mark. Theory Pract..

[96] Becker J.-M., Ringle C.M., Sarstedt M. (2018). Estimating moderating effects in pls-sem and plsc-sem: Interaction term generation*data treatment. J. Appl. Struct. Equ. Model..

[97] Hair J.F., Hult G.T.M., Ringle C., Sarstedt M. (2017). A Primer on Partial Least Squares Structural Equation Modeling (PLS-SEM).

[98] Brislin R.W. (1970). Back-translation for cross-cultural research. J. Cross-Cult. Psychol..

[99] Sánchez-Cañizares S.M., Cabeza-Ramírez L.J., Muñoz-Fernández G., Fuentes-García F.J. (2020). Impact of the perceived risk from COVID-19 on intention to travel. Curr. Issues Tour..

[100] Ross G.F. (1993). Destination evaluation and vacation preferences. Ann. Tour. Res..

[101] Chen C.-F., Tsai D. (2007). How destination image and evaluative factors affect behavioral intentions?. Tour. Manag..

[102] Chi C.G.-Q., Qu H. (2008). Examining the structural relationships of destination image, tourist satisfaction and destination loyalty: An integrated approach. Tour. Manag..

[103] Cronbach L.J. (1951). Coefficient alpha and the internal structure of tests. Psychometrika.

[104] Nunnally J.C., Bernstein I.H. (1994). Psychological Theory.

[105] Chin W.W., Newsted P.R. (1999). Structural equation modeling analysis with small samples using partial least squares. Stat. Strateg. Small Sample Res..

[106] Fornell C., Larcker D.F. (1981). Evaluating structural equation models with unobservable variables and measurement error. J. Mark. Res..

[107] Henseler J., Ringle C.M., Sarstedt M. (2015). A new criterion for assessing discriminant validity in variance-based structural equation modeling. J. Acad. Mark. Sci..

[108] Henseler J., Ringle C.M., Sinkovics R.R., Sinkovics R.R., Ghauri P.N. (2009). The use of partial least squares path modeling in international marketing. New Challenges to International Marketing.

[109] Chin W.W., Esposito V.V., Chin W.W., Henseler J., Wang H. (2010). How to Write Up and Report PLS Analyses. Handbook of Partial Least Squares.

[110] Reznik A., Gritsenko V., Konstantinov V., Khamenka N., Isralowitz R. (2020). COVID-19 Fear in Eastern Europe: Validation of the Fear of COVID-19 Scale. Int. J. Ment. Health Addict..

[111] Reza Jalilvand M., Samiei N., Dini B., Yaghoubi Manzari P. (2012). Examining the structural relationships of electronic word of mouth, destination image, tourist attitude toward destination and travel intention: An integrated approach. J. Destin. Mark. Manag..

[112] Fong L.H.N., Law R., Ye B.H. (2020). Outlook of tourism recovery amid an epidemic: Importance of outbreak control by the government. Ann. Tour. Res..

[113] Davies K. (2020). Festivals Post COVID19. Leis. Sci..

[114] Budd J., Miller B.S., Manning E.M., Lampos V., Zhuang M., Edelstein M., Rees G., Emery V.C., Stevens M.M., Keegan N. (2020). Digital technologies in the public-health response to COVID-19. Nat. Med..

[115] Serpas D.G., Ignacio D.A. (2021). COVID-19 fear mediates the relationship between perceived risk and preventive behaviors: The moderating role of perceived effectiveness. Psychol. Health.

[116] Yıldırım M., Özaslan A., Arslan G. (2021). Perceived risk and parental coronavirus anxiety in healthcare workers: A moderated mediation role of coronavirus fear and mental well-being. Psychol. Health Med..

